# Supplementary material for: Virtual patients as a tool for training pre-registration pharmacists and increasing their preparedness to practice: A qualitative study
Source: PLoS One. 2020 Aug 31;15(8):e0238226. doi: 10.1371/journal.pone.0238226 (PMC7458319; doi:10.1371/journal.pone.0238226)
Supplement: S1 File — (PDF) [file pone.0238226.s001.pdf]

Usefulness as a training tool

- What are your overall thoughts on the case studies/learning tool?
- Do you feel the VP/NI case studies were useful to your training? Why/why not?
- Did the VP/NI case studies help you learn/develop knowledge/skills?
    - Probe for specificity
- Do you think pre-registration training would benefit from the introduction of VP/NI case studies? Why/why not?
- What are your thoughts on using similar learning tools in the future?
    - Can link to training modules or prescribing courses

Support during pre-registration training

- Do you feel the VP/NI studies helped support your training? How?
- Could the VP/NI case studies have supported you in any other way? How?
- Did you use any case studies as evidence for competency sign off?
    - Probe for which ones, why/why not?
- Do you think the VP/NI case studies helped you feel more confident for the pre-registration exam or future practice? Why/why not?
- Do you feel you gained anything extra from completing the VP/NI case studies than if you hadn't had the opportunity?
    - Probe for specificity

Case study topics

- Which case studies do you feel were most useful? Why?
- Do you think the case studies impacted your learning? How?
- Are there are other topics you would have found useful to be turned into case studies?
    - Ask for examples and why they would be useful

Enjoyment

- Did you enjoy completing the VP/NI case studies? Why/why not?
- Did you feel more inclined to study from using the VP/NI case studies?
    - Engagement in learning/revision

OSCE preparation/alternative

- Did you have any OSCEs during pre-reg?
    - Did the cases help prepare you? Why/why not?
- If no OSCEs during pre-reg:
    - What are your thoughts on the case studies as an alternative?
- Did you have any other patient cases or problems to work through during pre-reg given to you by your training place? – How were these in relation to the VP/NI case studies?

Barriers

- Were there any barriers to using the VP/ NI case studies?
    - Probe for specificity
    - What could be done to overcome them

Improvements

- Are there any improvements that you think would make the VP/NI case studies a better training tool?
