# Supplementary material for: Virtual patients as a tool for training pre-registration pharmacists and increasing their preparedness to practice: A qualitative study
Source: PLoS One. 2020 Aug 31;15(8):e0238226. doi: 10.1371/journal.pone.0238226 (PMC7458319; doi:10.1371/journal.pone.0238226)
Supplement: S1 Table — Displays the frequency of topics suggested by trainees which they felt they could have had more support on during the pre-registration year. Each overall topic is subdivided into more specific topics which were reported by trainees. The ‘total number of participants’ illustrates the overall number of different participants who commented on that topic area. (PDF) [file pone.0238226.s002.pdf]

Topic Area		VP Group	NI Group
**OTC**	General	7	7
	Women's Health	1	0
	Skin	3	2
	Headlice	1	0
	Childhood conditions	1	0
	Gastrointestinal	0	1
	Ophthalmic	0	1
	Travel Health	0	1
	Hayfever	0	1
	Total no. of participants	7	9
**Clinical**	General	5	7
	Endocrine	3	4
	Respiratory	2	1
	Cardiovascular Disease	0	2
	IV Medications	0	2
	Hepatic	0	2
	Cytotoxic medicines	0	1
	Narrow Therapeutic Index medicines	0	1
	Palliative Care	0	1
	Central Nervous System	0	1
	Gastrointestinal	0	1
	Paediatrics	0	1
	Total no. of participants	9	7
**Services**	General	1	0
	Medicine Use Review (MUR)	3	0
	New Medicines Service (NMS)	2	0
	Smoking Cessation	1	1
	Medicines Optimisation	1	0
	Patient Group Directions (PGD)	1	0
	Total no. of participants	5	3
**Professional Practice**	Law	2	2
	Complaints	1	0
	Controlled Drugs	1	0
	Fitness to Practice	1	0
	Substance Misuse	0	1
	Total no. of participants	3	2
**Skills**	Interdisciplinary	5	5
	Using resources	1	0
	Extemporaneous dispensing	1	0
	Drug Interactions	0	1
	Calculations	0	1
	Total no. of participants	5	6
