# Supplementary material for: Virtual patients as a tool for training pre-registration pharmacists and increasing their preparedness to practice: A qualitative study
Source: PLoS One. 2020 Aug 31;15(8):e0238226. doi: 10.1371/journal.pone.0238226 (PMC7458319; doi:10.1371/journal.pone.0238226)
Supplement: Attachment — Submitted filename: Response to Reviewers.docx [file pone.0238226.s003.docx]

Academic Editor Comments

 Please ensure that your manuscript meets PLOS ONE's style requirements, including those for file naming

The PLOS ONE style templates were reviewed and article/figures/supplementary files amended appropriately.

Please include a copy of the interview guide used in the study, in both the original language and English, as Supporting Information, or include a citation if it has been published previously.

Interview guide has been included as Supplementary File 1.

Reviewer #1 Comments

This exploration of online tools for developing educational materials to pharmacy trainees is potentially interesting and relevant to those in the field, but not necessarily as portrayed by the authors. Although the paper does not stand up well as is, a re-framing and appropriate revision of the discussion and conclusions might make this paper more defensible and, potentially, more interesting.

The premise of this paper is reasonable to start with. Innovative approaches to providing training to pharmacy students are certainly worthy of research and exploration, and interactive avatars seem worth a try, particularly if they haven't been used before. The juxtaposition of the avatars and a non-interactive version is a good
step, allowing for the possibility of comparative analysis.

Confusion in my reading of the methods leave me with significant concerns regarding the validity of some of the claims made in the paper.

- As I understand the methods, this study was primarily a between-subjects study, with participants using either the avatar-based or the non-interactive interfaces. My confusion hinges on the cryptic comment "At the end of the three months, participants who completed all three cases were provided with the comparative case studies and invited via email to participate in a telephone interview." I do know that this last phrase means but it seems particularly important

The phrase was amended to remove any links to the case studies being comparable. Detail was added to ensure the phrase was clearer regarding the opportunity for pre-registration trainees to use the other style of case study as a learning tool.

- If each participant only saw one interface, any comparative comment about liking one interface more than the other (there are several such comments in the manuscript) would be completely invalid, as no participant would have any basis for comparison – Comments which relate to comparisons between the two learning tools have been removed throughout the article. An interview guide was created which asked questions on the same topics, however any claims to a comparison of the learning tools have been removed; except where trainees *did* use both of the tools and provided such comments. However it is made clear that this only relates to a small proportion of the participants.
- If this comment is to be interpreted to mean that participants saw both versions, there might be some basis for comparison, but as we have no information about how long each participant spent with the second interface, we have no way to judge the validity of any comparisons.
  Specific comments relating to comparisons have been removed. Information regarding how long participants had access to the learning tools has been added to the article. Participants were able to access each case study for one month sequentially, to give a total intervention period of three months.
- Furthermore, we are later told that only three participants looked at both interfaces, which effectively eliminates any hope of direct comparison. These methods need to be clarified, and any comparisons between the two interfaces need to be justified.
  It has been clarified that all trainees were given the opportunity to use the other type of learning tool. Only four trainees reported that they had done and provided comments as such. Specific comments relating to comparisons have been removed and made clear when the participants had used both types of learning tool.
- Even if these changes are made, I am concerned about inappropriate quantitative comparisons between the designs. From the abstract: "Pre-registration trainees appeared overall to prefer learning via the interactive clinical avatars over the non-interactive case studies and they were associated with greater self-reported development of knowledge, skills and confidence for practice." This is a quantitative claim that should be accompanied by measurements and statistical
  tests. Barring that, these claims should be removed.

Any statements which could be seen as quantitative claims have been rewritten to appropriately report qualitative comments.

- I have no beef with the qualitative analysis or the thematic discussion, but I was struck by how minor the differences between the designs appear to be in the end. This might in itself be noteworthy - perhaps the realistic content is really what's important here?
  More information has been added into the manuscript regarding the realism of the virtual patient. Also, more detail which relates to the non-interactive cases being above what trainees would normally have has been added.
- I suggest clarifying the methods and avoiding any claims that imply direct comparison or quantitative analysis.
  Amended throughout as noted above.

Other minor notes:

1.The first paragraph in the introduction is a bit of a mess. Consider focusing the thinking to make a stronger introduction.
Paragraph 1 of the introduction has been amended.

2. "Pre-registration" is a very specialized term that might not have any meaning to readers outside of the field. Suggest removing that phrase and substituting "pharmacist trainees" or some other alternative.
Pre-registration pharmacist training is explained in paragraph 1 of the introduction. It was felt important to use this term to distinguish between undergraduate pharmacist trainees where the majority of previous research has been focused and postgraduate phamacists trainees who may be qualified pharmacist undertaking a further postgraduate course (e.g. prescribing).

3. I have no beef with telephone interviews per se, but in the 2020 world of ubiquitous web conference and screen sharing, citing a 1995 article in support of telephone calls seems a bit anachronistic. Why not use web conferencing?
A more up-to-date reference has been added to the article. Telephone interviews were used as they were identified as the least unobtrusive method. Some of the interviews took place during participants’ working hours and they could easily use a phone. However, more technologically advanced methods would be considered in future research.

4. More details are needed on the construction of the avatar-based training. This was clearly more than "standard HTML" How were the avatars built?
More information has been added into the article. The page layout was defined using HTML with CSS to define the position and colour of screen elements. For the VP cases using free-text variations, once the 'speak to patient' button was clicked the text was sent to a web service hosted on the same server to process the text. This then returned a code to the site which was processed by the client side Javascript (using the jQuery library) to determine what animation to play and how the case should behave next. The MCQ cases processed everything client side, skipping the web service step.

5. The 38 topic areas identified in the interviews should be provided along with the paper. This is potentially useful information.
A table showing the topic areas has been included as Supplementary File 2.

Reviewer #2:

Strengths
* The work is well motivated, well written, and describes a well conducted research
* The authors are familiar with the literature and do not overstate claims (they are aware of work in medicine and nursing that has established similar findings over a decade ago in some cases)
* The findings are valuable to the pharmacology education community

Weaknesses

- The findings already mirror what has been found in other domains some time lack so have limited applicability to advancing virtual patient design/research. The results might only be new information to pharmacology education researchers.
  It is agreed that the findings are similar to previous research in other healthcare professions. The article has tried to infer the greater advancement this brings to the pharmacy profession and has focused on the use of free-text virtual patients which are under-researched in the literature.
- Incorrect terminology is used throughout. Please review closely with domain experts:
  - Agents vs avatars. what you are using are "agents" - virtual humans controlled by algorithms, not "avatars" virtual humans controlled by humans. avatars and virtual humans are not synonyms
    Terminology changed to ‘virtual patients’ throughout.
  - Markov model - you can't have a "markov model decision tree", they mean very different things. avoid using terminology that is not well understood.
    Markov model removed.
- Animations were encoded as mp4 files - this needs much more clarification as mp4 files are a compression format and how animations are encoded into them is unclear.
  More information has been added into the article. Characters were modelled, textured and animated using Autodesk's Maya 3D package. Once animated, a series of still images were rendered using the rendering package Mental Ray supplied with Maya. The still images were then composited using Adobe After Effects to create the final .mp4 animation files.
- It is unclear if the user selected conversation choices (e.g. EHC case) or had a free text opportunity (child illness case)
  How the user interacted with each virtual patient simulation has been added into the methods section and it has been made clear which utilised multiple-choice vs free-text input.
- Virtual patients are now common and thousands of medical and nursing curricula with many commercial systems being used around the world

This is true. The differences of this virtual patient system (free-text, animated feedback) has been added into the manuscript. Implications for healthcare training have been made clearer.

- The discussion about limitation about JT interviewing the participants needs to be rewritten. You can't say that you tried to not be biased and that you believe the answers were candid, because fundamentally you can't know that. Rephrase to acknowledge the limitation and highlight improvements for future work.
  The limitation regarding JT interviewing the participants has been rewritten.

Minor

- This document could use a good edit for grammar errors
  A thorough proofread has been done and edited where appropriate.
