# Supplementary material for: Virtual patients as a tool for training pre-registration pharmacists and increasing their preparedness to practice: A qualitative study
Source: PLoS One. 2020 Aug 31;15(8):e0238226. doi: 10.1371/journal.pone.0238226 (PMC7458319; doi:10.1371/journal.pone.0238226)
Supplement: Attachment — Submitted filename: Response to Reviewers.docx [file pone.0238226.s004.docx]

Thank you for your review of my manuscript. I have addressed your comments below.

1. My main conceptual concern is that your study essentially introduces two interlinked interventions: 1. The use of the conversational agent, and 2) the use of the visual avatars. It is possible that both of these are needed to have a strong impact on educational experiences - this study is not designed in a way that would answer that question. However, I would like to see something in the discussion about the possibility that the interactive component on its own might or might not be as useful as the virtual patients described in this paper.

- This point is acknowledged and agree that the research was not designed in a way to separate the impact of the conversation agent and visual avatar. The manuscript has been amended (line 485-488) “This research was not designed in a way to separate the impact of the interactive component and visual avatar; this warrants further research to determine how these elements can affect learning when used separately versus in combination.”

2. The term "pre-registration" is still used in the abstract in a manner that I found confusing. If this were a journal that was (a) focused on pharmacy education, and (b) focused on a British audience, this usage would be acceptable, but as neither of these things are true, this language obscures rather than clarifies. I suggest revising to "pre-certification", internship or something similar.

- The abstract has been amended to clarify what pre-registration training means. Line 20-22 “In the United Kingdom, pre-registration training refers to a year of workplace based training which pharmacy graduates must complete prior to professional registration as pharmacists.”

3. The abstract refers to the "framework approach". I assume this is referring to the five-stage framework approach used for qualitative analysis? This should be clarified.

- This has been clarified in the abstract – line 28.

4. The abstract makes this significant claim: "however those who used the virtual patient commented on the development of real-life complex skills and aspects of learning, and those who used the non-interactive cases focused on more simple skills and knowledge." I don't see this justified in the presentation of the results or the discussion. I suggest reframing.

- The abstract has been amended (lines 34-37) “those who used the virtual patient commented on the development of communication skills and an increase in confidence for practice and those who used the non-interactive cases focused on knowledge acquisition and numeracy”. The discussion has also been amended to reflect the changes (lines 407-409).

5. Under "Participants", please clarify what is meant by "sector of training".

- This has been clarified on line 107 (community or hospital pharmacy).

6. Line 160-161. I presume MCQ means "multiple-choice question" , but it is not defined, and there is no indication of which of the scenarios involved this type of question.

- Line 162-163 has been amended to ‘multiple–choice input cases’. The explanation of which cases used which input style was provided previously: lines 145-146 “The EHC simulation utilised both multiple-choice input and free-text input”, line 150 “They were able to interact with the VP via multiple-choice input”, line 151 “The childhood illness VP simulation utilised free-text input”.

7. Results. 20 interviews were conducted, but earlier we were told that 56 participants were eligible. Did 39 decline? this should be clarified.

- The manuscript has been amended (line 214-215) “The remaining 36 participants who were invited for a telephone interview did not respond to the invitation.”

8. The gender differential (heavily skewed toward female in both cases) should be mentioned in the discussion as having a possible impact on the observations.

- The manuscript has been amended (lines 491-493) “Although the participants in both groups were mostly female and could have impacted the data obtained, this is consistent with the distribution profile of pharmacists in practice [48].”
